# Significance of Magnetic Resonance Imaging–Assessed Tumor Response for Locally Advanced Rectal Cancer Treated With Preoperative Long-Course Chemoradiation

**DOI:** 10.1200/JGO.2015.001479

**Published:** 2016-02-10

**Authors:** Mohamed Salah Fayaz, Gerges Attia Demian, Wael Moftah Fathallah, Heba El-Sayed Eissa, Mustafa Shawki El-Sherify, Sadeq Abozlouf, Thomas George, Suzanne Mona Samir

**Affiliations:** All authors, Kuwait Cancer Control Center, Shuwaikh, Kuwait.

## Abstract

**Purpose:**

To study the predictive and prognostic value of magnetic resonance imaging (MRI)–assessed tumor response after long-course neoadjuvant therapy for locally advanced rectal cancer.

**Methods:**

This study included 79 patients who had T3 or T4 and/or N+ rectal cancer treated with long-course neoadjuvant chemoradiation. MRI-assessed tumor regression grade (mrTRG) was assessed in 64 patients. MRIs were reviewed by the study radiologist. Surgical and pathologic reports for those who underwent surgery were reviewed. Disease-free survival (DFS) was estimated. Progression during therapy, local relapse, metastasis, and death resulting from the tumor were classified as events. Statistical significance was calculated.

**Results:**

In 11 patients, the tumor completely disappeared on MRI; that is, it had an mrTRG of 1. All but one patient, who chose deferred surgery, had a complete pathologic response (pCR), with a positive predictive value of nearly 100%. Of the 20 patients who had an mrTRG of 2 on MRI, six had a pCR. mrTRG 3, mrTRG 4, and mrTRG 5 were detected in 24, six, and three patients, respectively, of whom only one patient had a pCR. The 2-year DFS was 77%. The mrTRG was significant for DFS. The 2-year DFS was 88% for patients with a good response versus 66% for those with a poor response (*P* = .046).

**Conclusion:**

MRI-assessed complete tumor response was strongly correlated with pCR and, therefore, can be used as a surrogate marker to predict absence of viable tumor cells. Our results can be used to implement use of mrTRGs in larger prospective correlative studies as a tool to select patients for whom deferred surgery may be appropriate. Also, those with a poor response may be offered further treatment options before definitive surgery.

## INTRODUCTION

The current standard of care for patients with T3 or T4 and/or node-positive rectal adenocarcinoma is to offer preoperative concomitant chemoradiotherapy (CRT) followed by total mesorectal excision (TME). The effect of tumor downstaging becomes increasingly important. The German Rectal Cancer Study investigators found that the most important independent prognostic factors in multivariable analysis for disease-free survival (DFS) are pathologic T category and nodal status after preoperative treatment.^1^ Dworak et al showed that the 5-year DFS rate was significantly associated with the tumor regression grade (TRG).^2^ TRG was determined as the amount of viable tumor versus fibrosis. Therefore, TRG 0 was defined as complete absence of fibrosis, whereas TRG 4 showed no viable tumor cells. The 5-year DFS rate was 86% for TRG 4, 75% for TRG 2 + 3, and 63% for TRG 0 + 1 (*P* = .006).

High-resolution magnetic resonance imaging (MRI) has been used to assess tumor response before surgical resection. By applying the principles of histopathologic TRG and by exploiting the characteristic MRI low-signal–intensity appearances of fibrosis, it has been possible to develop a similar MRI-based TRG system. The MRI-assessed TRG (mrTRG) was found to be an independent prognostic factor for overall survival (OS) and DFS.^3^

Complete pathologic response (pCR) after CRT has led to the proposal of a nonoperative approach as an alternate treatment for highly selected patients with a complete clinical response (CR). Habr-Gama et al reported findings from 99 patients with a clinical CR who were treated with observation alone. The 5-year OS and DFS rates were 93% and 85%, respectively.^4^

Our aim was to study the predictive and prognostic value of MRI-assessed tumor response after long-course neoadjuvant therapy for locally advanced rectal cancer treated in our center.

## METHODS

### Patients

Between March 2008 and December 2012, 79 consecutive patients with a diagnosis of locally advanced rectal cancer at Kuwait Cancer Control Center, Kuwait, were considered for inclusion in this retrospective study. Inclusion criteria consisted of biopsy-proven rectal adenocarcinoma, locally advanced disease staged on MRIs as T3 to 4 and/or N+, and neoadjuvant treatment consisting of a long course of preoperative CRT given as 50.4 Gy of radiation plus 2 × 825 mg/m^2^/day of capecitabine (Xeloda; Hoffmann-La Roche, Basel, Switzerland). Exclusion criteria were metastatic disease and neoadjuvant treatment consisting of a short course of 5 Gy radiation on 5 consecutive days.

### MRI

MRI was performed at 1.5 T by using a phased-array body coil. Patients underwent pretreatment pelvic MRI for tumor staging and restaging MRI for assessment of response 6 to 8 weeks after completion of neoadjuvant CRT. The imaging protocol consisted of the following: standard T2-weighted, fast spin-echo sequences in the three orthogonal directions and an axial diffusion-weighted sequence with background body-signal suppression and *b* values of 0, 50, 100, 500, 750, and 1,000 sec/mm^2^. The axial T2- and diffusion-weighted imaging sequences were angled in a plane perpendicular to the axis of the tumor, as defined on sagittal images. The coronal T2-weighted plane was angled parallel to the tumoral axis.

Primary tumor response was manifested as either replacement of the signal of the tumor by low-signal-intensity fibrosis or the development of mucin pools representing tumor necrosis. Residual tumor was interpreted as any area of persistent intermediate signal intensity on high-resolution images.

Nodal stage was based on the interpretation of the lymph node border and signal intensity. A node was considered positive if an irregular border or mixed signal intensity was demonstrated.

### mrTRG

mrTRG 1 was identified as the absence of any tumor signal. mrTRG 2 was identified as small amounts of residual tumor visible but with a predominant fibrotic area of low signal intensity. mrTRG 3 was identified as mixed areas of low-signal-intensity fibrosis and intermediate signal intensity present but without predominance of tumor. mrTRG 4 was identified by predominantly tumor signal intensity remaining, with minimal fibrotic low signal intensity. mrTRG 5 was identified as no fibrosis evident and only tumor signal intensity visible.^3^

### Radiotherapy

Radiotherapy was given by means of a conformal three-field technique to deliver 45 Gy in 25 fractions over 5 weeks to the true pelvis and 5.4 Gy in three fractions as a tumoral boost. Concomitant capecitabine was given at a dose of 825 mg/m^2^ twice daily during radiotherapy days. Re-evaluation MRI was done after an average of 6 weeks after the completion of radiotherapy. Surgery in the form of TME with or without sphincter preservation was usually scheduled within 8 to 9 weeks after the completion of radiotherapy. Adjuvant chemotherapy (ACT) was given according to the patient's and tumor's characteristics and at the discretion of the patient and the medical oncologist. No patients received postoperative radiotherapy.

### Data and Statistical Analysis

mrTRG was assessed in 64 patients. MRIs were reviewed by the study radiologist. Surgical and pathology reports for those who underwent surgery were reviewed. DFS was estimated by using the Kaplan-Meier method. Progression during therapy, local relapse, metastasis, and death resulting from the tumor were classified as events. Statistical significance was calculated by using the log-rank test and defined as *P* ≤ .05.

## RESULTS

The median age was 53 years, and 60% of patients were male (Table 1). The majority presented with T3 (78.5%) and N2 (61%) disease. The median tumor size was 6 cm (range, 2.5 to 12 cm). The median distance from the anal verge was 5 cm (range, 1 to 14 cm). The pretreatment carcinoembryonic antigen level was available for 58 patients. It was normal in 41 and elevated in 17 patients (range, 5 to 102 ng/mL). All patients received preoperative, long-course CRT with concomitant capecitabine. Six patients were lost to follow-up after their completion of preoperative CRT, and three refused surgery.

**Table 1:**
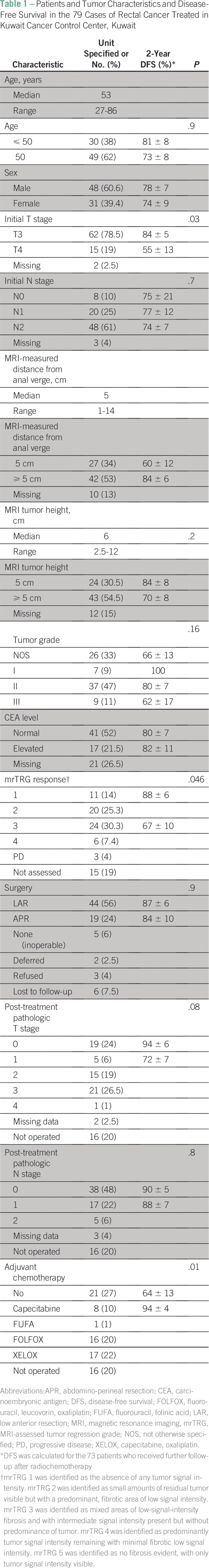
Patients and Tumor Characteristics and Disease-Free Survival in the 79 Cases of Rectal Cancer Treated in Kuwait Cancer Control Center, Kuwait

Staging [^18^F]fluorodeoxyglucose positron emission tomography/computed tomography was done for 31 patients. All had a hypermetabolic rectal lesion with a median standardized uptake value of 14 (range, 4 to 43). Of these, 17 patients underwent a positron emission tomographic evaluation of response. Metabolic response was seen in 15 patients, with a median reduction of the standardized uptake at 45% of its initial value.

MRI evaluation of response was available for 64 patients. Median timing of MRI evaluation was 38 days (range, 23 to 88 days) after completion of CRT. Eleven patients had complete regression of the tumor on MRI, or mrTRG 1. All but one, who chose deferred surgery, had pCR, with a positive predictive value of nearly 100%. Of the 20 patients who had mrTRG 2, six had pCR. mrTRG 3, mrTRG 4, and mrTRG 5 were detected in 24, six, and three patients, respectively, of whom only one patient had pCR.

Five patients proved to have inoperable disease at the time of surgery: two underwent exploration and had peritoneal disease, two had radiologic systemic progression, and one had still-locally fixed disease. Two patients chose deferred surgery. Sixty-three patients had evaluable postoperative pathology.

pCR of the primary tumor was detected in 19 patients. Two of them had residual lymph node disease (10.5%), and 17 had negative lymph nodes. Among the other 42 patients with residual primary disease, 19 had positive lymph nodes (45%). The median number of dissected nodes was 11 (range, 0 to 34).

ACT was used in 42 patients. An oxaliplatin-based regimen was used in 33 of them. After a median follow-up of 25 months (range, 4 to 68 months), 15 patients had disease progression. Nine developed systemic disease progression, five had persistent disease, and three had isolated local recurrence; one of the three had an anastomotic recurrence that was re-excised completely, and one had recurrence after deferred surgery.

The DFS rate was calculated for the 73 patients who received further follow-up after CRT. The DFS rate for the 73 patients was 77% ± 6%. DFS was significantly worse in those who presented with T4 disease versus T3 disease (*P* = .03). It was lower in patients with an MRI-assessed poor response (mrTRG 3-5), that is, 66% versus 88% for those with a good response (mrTRG 1 or 2), with a *P* value of .046. Also, patients who received ACT had DFS significantly better than that of those who did not (*P* = .01).

The 2-year local recurrence–free survival rate was 94% ± 3%, specifically 92% ± 5% for mrTRG 1 and mrTRG 2 and 97% ± 3% for mrTRG 3-5 (*P* = .6). It was 100% for mrTRG 1 and 93% ± 4% for the others. The 2-year distant metastasis–free survival was 83% ± 5%: 95% ± 4% for good responders and 75% ± 9% for poor responders (*P* = .03).

## DISCUSSION

Our study was designed to evaluate the value of MRI assessment of tumor response to neoadjuvant CRT and its effect on outcomes for our patients. The strengths of the study were that all the patients received a uniform treatment regarding the radiotherapy dosage and fractionation and the chemotherapy sensitizer. Also, the pretreatment and post-treatment MRIs were retrospectively reviewed by the study radiologist, who was blinded to the surgical pathology data. The limitations are that it was a retrospective study, that only 80% of patients underwent MRI assessment and 80% received surgery, 7.5% were lost to follow-up, and that surgical pathology data were not available for 4% of patients.

Despite these limitations, our data show that findings from MRI assessment of TRG after preoperative therapy are predictive of DFS. The MERCURY study investigators were the first to show that mrTRG is predictive of both DFS and OS and it gives prognostic information regarding the risk of local recurrence.^3^ The relationship between pathologic TRG and outcome has been documented in many studies.^1,2,5^ Last, meta-analysis of data from 3,105 patients in 14 studies, of 484 patients had pCR, showed that patients with pCR had significantly better DFS and OS with lower risk of LR and distant metastasis (*P* < .001) and the benefit was confirmed on multivariate analysis with a hazard ratio of 0.54 (range, 0.4 to 0.73) for DFS and 0.65 (range, 0.7 to 0.89) for OS.^6^

Another important finding in our study was that MRI-depicted complete tumor regression that was highly predictive of pCR of the primary tumor. This is also supported by findings from the MERCURY investigators, who showed that mrTRG was statistically correlated with post-treatment pathologic T stage, or ypT.^3^ Therefore, MRI-detected CR may be used as a marker to select patients who may be candidates for a watchful policy, especially those who may lose sphincter control with surgery. Habr-Gama et al reported that, among 90 patients who had complete clinical remission and who were offered the wait-and-watch policy, local recurrence developed in 31%. Salvage therapy was possible in > 90% of recurrences, leading to 94% local disease control, with 78% organ preservation.^7^ Despite the impressive results of intensive wait and watch in patients who achieve clinical CR, surgery is still the standard of care.

Given the findings from a phase II nonrandomized trial^8^ that showed an increased proportion of pCR with the addition of mFOLFOX6 between CRT and surgery, Garcia-Aguilar et al are conducting a large phase II randomized trial^9^ to test the hypothesis that the addition of induction or consolidation chemotherapy to CRT and TME or nonoperative management would improve 3-year DFS compared with the standard CRT, TME, and ACT. They aim to increase the percentage of patients who may potentially benefit from nonoperative care.

Nonoperative management is particularly attractive in our patients of Arab descent, who often present at a relatively young age. Although the incidence of colorectal cancer (CRC) in the Arab population is lower than that in Western groups (the age-standardized incidence rate being 11/10^5^ in Kuwait^10^ and 6/10^5^ in Egypt^11^), two negative epidemiologic features are observed. First, the incidence of CRC at ages younger than 40 years is relatively high.^11^ Second, a high percentage of CRCs is located in the rectum: 37% in Egypt^11^ and Kuwait^10^ compared with 27% in the United States^12^ and 29% in the United Kingdom.^13^ To secure a better quality of life, by taking into account sexual function and sphincter competence, these young patients often request nonoperative care.

In our study, the probability of positive lymph nodes with pCR of the primary tumor was 10.5%, whereas positive lymph nodes were detected in 45% when pCR was not achieved for the primary disease. However, none of the patients who had MRI-detected CR had pathologically positive lymph nodes. These results were in concordance with those of Rödel, et al, who found that pCR of the primary tumor was associated with 10% post-treatment pathologic N stage, or ypN, positivity. In contrast, patients with tumors showing intermediate tumor regression had a 32% risk of post-treatment pathologic N stage positivity, and poor tumor regression was associated with a 42% incidence of nodal involvement.^1^

One of the shortcomings of our study was the short-term follow-up. However, in a 10-year follow-up study of 297 consecutive patients who received CRT, of whom 44 patients (15%) had pCR, only one patient had disease recurrence, and no recurrence happened after the first 2 years of follow-up.^14^

In conclusion, MRI-assessed complete tumor response was strongly correlated with pCR and therefore can be used as a surrogate marker to predict absence of viable tumor cells. Our results can be used to implement mrTRG in larger, prospective correlative studies, as a tool to select patients for whom deferred surgery may be appropriate. Assessment of mrTRG can be used to predict the DFS outcome so that patients with a poor response may be offered further treatment options before definitive surgery.
